# Endocrine-informed monitoring of scoliosis in Prader–Willi syndrome: integrating neuroendocrine pathophysiology, growth hormone therapy, and pubertal transition

**DOI:** 10.3389/fendo.2026.1803919

**Published:** 2026-04-13

**Authors:** Minshun Zhu, Qiang Sun, Jian Zhang, Kuo Cai, Zhipeng Guo, Qing Wu, Yueling Wang, Jiaping Chen

**Affiliations:** Department of Rehabilitation, Lu’an Hospital of Traditional Chinese Medicine, Lu’an, China

**Keywords:** endocrine monitoring, growth hormone therapy, Prader-Willi syndrome, puberty, risk stratification, scoliosis

## Abstract

Prader–Willi syndrome (PWS) is a neuroendocrine disorder characterized by hypothalamic dysfunction, congenital hypotonia, abnormal growth trajectories, and impaired pubertal development, all of which contribute to a markedly increased risk of scoliosis, with a cumulative prevalence reaching up to 70–80% by skeletal maturity, significantly exceeding that of idiopathic scoliosis. Unlike idiopathic scoliosis, spinal deformity in PWS follows a distinct bimodal pattern, with critical vulnerability during infancy and a second acceleration during pubertal transition. Growth hormone (GH) therapy, a cornerstone of PWS management, substantially improves linear growth, body composition, and muscle strength, yet its relationship with scoliosis onset and progression remains a clinical challenge due to the potential for accelerated growth during critical developmental windows, which may unmask or exacerbate underlying spinal instability. Current scoliosis surveillance strategies in PWS are largely extrapolated from idiopathic scoliosis and fail to account for the unique neuroendocrine and biomechanical context of this syndrome. In particular, endocrine modifiers such as GH treatment status, growth velocity, hypogonadism, pubertal stage, body composition, and genotype-specific phenotypes are rarely integrated into structured monitoring protocols. In this narrative review, we synthesize epidemiological, mechanistic, and clinical evidence to elucidate the neuroendocrine and biomechanical pathways underlying scoliosis development in PWS, including the roles of hypotonia-related instability, altered vertebral growth modulation, and delayed epiphyseal maturation. We critically examine the dualistic effects of GH therapy, the impact of pubertal maturation, and genotype–phenotype associations as key determinants of scoliosis risk and progression. Building on this evidence, we propose an endocrine-informed, risk-stratified scoliosis monitoring framework that integrates growth dynamics, hormonal status, body composition, and spinal parameters to guide surveillance intensity, imaging strategies, and multidisciplinary referral. By shifting the focus from isolated curve detection to longitudinal, endocrine-guided surveillance, this review provides a clinically actionable model to optimize early identification and management of scoliosis in children and adolescents with PWS. This framework aims to support coordinated endocrine–orthopedic care and inform future prospective studies designed to refine outcome measures and ultimately improve long-term musculoskeletal and quality-of-life outcomes in this vulnerable population.

## 1 Introduction

Prader–Willi syndrome (PWS) is a rare neuroendocrine disorder caused by the loss of expression of paternally inherited genes on chromosome 15q11.2–q13. Hypothalamic dysfunction underlies its characteristic clinical phenotype, which includes congenital hypotonia, abnormal growth trajectories, hyperphagia with progressive obesity, hypogonadism, and multiple pituitary hormone deficiencies. In addition to metabolic and developmental complications, individuals with PWS exhibit a markedly increased burden of musculoskeletal disorders, among which scoliosis represents one of the most prevalent and clinically significant challenges. Reported prevalence rates range from approximately 15% to over 80%, with cumulative incidence increasing across childhood and adolescence and reaching up to 70% by skeletal maturity, particularly during periods of rapid growth (1). This wide range reflects variations in cohort age, diagnostic criteria (e.g., Cobb angle threshold), and surveillance intensity across studies. Importantly, scoliosis in PWS is not merely a list of risk factors but arises from their mechanistic interaction: congenital hypotonia compromises baseline spinal stability, while obesity alters biomechanical loading. Furthermore, the spinal deformity in PWS is not confined to the coronal plane; a significant proportion of patients present with kyphoscoliosis, where coronal curvature is accompanied by increased thoracic kyphosis, adding morbidity from sagittal imbalance (2). These factors are then exacerbated by Growth hormone (GH)-induced growth acceleration and the atypical hormonal milieu of puberty, collectively modulating vertebral growth and progression risk in three dimensions.

Scoliosis in PWS differs fundamentally from adolescent idiopathic scoliosis in both natural history and pathophysiology. The condition demonstrates a bimodal age distribution, with early-onset curves often emerging in infancy in association with severe hypotonia and neuromuscular imbalance, and a second peak during adolescence coinciding with accelerated linear growth (3, 4). This bimodal distribution is critical, as it distinguishes early-onset scoliosis (EOS, onset <10 years), driven primarily by profound hypotonia and neuromuscular weakness, from adolescent-onset scoliosis, which shares some features with AIS but occurs in the unique endocrine context of PWS. Consequently, the monitoring and management paradigms for these two groups should be informed by different orthopedic frameworks (3, 5). Altered body composition, reduced muscle strength, impaired postural control, and obesity-related mechanical loading collectively compromise spinal stability in PWS and contribute to heterogeneous progression patterns, ranging from stable mild curves to rapidly progressive deformities during growth spurts. These features underscore the limitations of directly extrapolating idiopathic scoliosis surveillance strategies to the PWS population.

GH deficiency is highly prevalent in PWS, and recombinant human GH therapy has become a cornerstone of management due to its well-established benefits on linear growth, body composition, muscle strength, and overall health (6). However, GH therapy also accelerates growth velocity, particularly during treatment initiation and pubertal transition, raising long-standing concerns regarding its potential impact on scoliosis onset and progression (7). While early case reports raised concern, larger longitudinal studies have not confirmed a causative role, suggesting that observed progression during GH therapy may reflect the natural history of PWS intersecting with periods of rapid growth, rather than a direct adverse effect (8). Pubertal development in PWS is frequently delayed, incomplete, or medically induced as a consequence of hypogonadism, although substantial interindividual variability exists. Given the critical role of sex steroids in skeletal maturation and muscle development, the interaction between GH-induced growth acceleration and atypical pubertal progression creates a period of heightened vulnerability for scoliosis progression during adolescence (4).

Despite the high prevalence and complex endocrine determinants of scoliosis in PWS, current surveillance strategies remain largely extrapolated from idiopathic scoliosis paradigms and are insufficiently adapted to the syndrome’s unique neuroendocrine context. Endocrine modifiers such as growth velocity, GH treatment status, pubertal stage, body composition, and genotype-specific phenotypes are rarely integrated into structured monitoring protocols. In addition, scoliosis surveillance is often fragmented across specialties, with limited coordination between endocrine and orthopedic care, potentially delaying recognition of rapid progression during high-risk developmental windows (1). This ‘endocrine-blind’ approach may lead to suboptimal timing of surveillance and delayed intervention.

In this narrative review, we synthesize epidemiological, mechanistic, and clinical evidence to reframe the understanding and monitoring of scoliosis in PWS within an endocrine-informed framework. We critically evaluate the roles of GH therapy and pubertal development as dynamic modifiers of scoliosis risk and propose a risk-stratified monitoring framework that integrates growth dynamics, hormonal status, pubertal stage, body composition, and baseline spinal parameters. By shifting the focus from isolated curve detection to longitudinal, developmentally informed surveillance, this review aims to support coordinated endocrine–orthopedic care and inform future research designed to optimize musculoskeletal and quality-of-life outcomes in children and adolescents with PWS.

## 2 Epidemiology and natural history of scoliosis in PWS

Scoliosis is one of the most prevalent musculoskeletal complications in individuals with PWS, with reported prevalence rates ranging from approximately 15% to over 80% across published cohorts. This wide variability reflects differences in age distribution, genetic subtype composition, diagnostic criteria, and intensity of surveillance among studies. Large cohort analyses have demonstrated that scoliosis prevalence increases progressively with age, underscoring its cumulative nature in PWS. In a cohort of 180 genetically confirmed patients, the overall point prevalence of scoliosis reached 83.3%, affecting 80.2% of children and adolescents and 87.8% of adults, highlighting the persistence and progression of spinal deformities across the lifespan (9). Longitudinal observations further suggest that the cumulative incidence of scoliosis reaches up to 70% by skeletal maturity, emphasizing the need for sustained surveillance throughout growth and development (10).

The phenotypic presentation of scoliosis in PWS is heterogeneous and differs from idiopathic scoliosis. Early-onset scoliosis frequently emerges during infancy or early childhood and is often associated with severe hypotonia and neuromuscular imbalance. These early curves typically present as long, smooth “C-shaped” deformities in the coronal plane and may initially be flexible or postural in nature. Notably, the sagittal profile is also commonly affected, with a significant prevalence of increased thoracic kyphosis, leading to a kyphoscoliotic pattern that further compromises spinal balance and pulmonary function (3, 5, 11). In contrast, scoliosis developing later in childhood or adolescence often resembles adolescent idiopathic scoliosis in curve pattern but arises in the context of distinct biomechanical and endocrine abnormalities characteristic of PWS. Importantly, the natural history of scoliosis in PWS follows a bimodal distribution, with two critical periods of increased risk: an early peak during infancy related to profound hypotonia and delayed motor development, and a second, more prominent peak during puberty, when rapid linear growth accelerates curve progression (1, 12). While precise proportions vary, studies suggest that a significant minority of patients present with early-onset scoliosis in the first decade of life, while the majority of curves are identified or progress during the adolescent growth spurt.

Disease progression in PWS-associated scoliosis is highly variable between individuals. Some curves remain stable for prolonged periods, whereas others demonstrate rapid deterioration, particularly during phases of accelerated growth. Multiple modifying factors appear to influence this variability, including genetic subtype, body composition, bone health, and exposure to GH therapy. Several studies have suggested that patients with paternal deletions may exhibit a higher prevalence of scoliosis compared with those with maternal uniparental disomy, although this association has not been consistently observed across cohorts (13). Similarly, the relationship between body mass index (BMI) and scoliosis risk in PWS remains complex and somewhat complex and non-linear; while obesity is a hallmark feature of the syndrome, some reports suggest that higher BMI may be associated with a lower prevalence or severity of scoliosis, potentially reflecting mechanical or muscle-related factors, though obesity may complicate clinical management and surgical outcomes (3).

The potential influence of GH therapy on the epidemiology and progression of scoliosis in PWS has been a subject of long-standing debate. Longitudinal studies with follow-up periods of up to eight years have shown that GH therapy does not significantly increase the prevalence or severity of scoliosis compared with untreated controls, despite marked improvements in linear growth and body composition (14, 15). These findings suggest that scoliosis progression observed during GH treatment likely reflects the natural history of the disorder intersecting with periods of rapid growth rather than a direct adverse effect of therapy. Notably, bone mineral density has been reported to correlate inversely with scoliosis severity, indicating that skeletal health may act as an important modifier of curve progression in this population (14).

**Figure 1:**
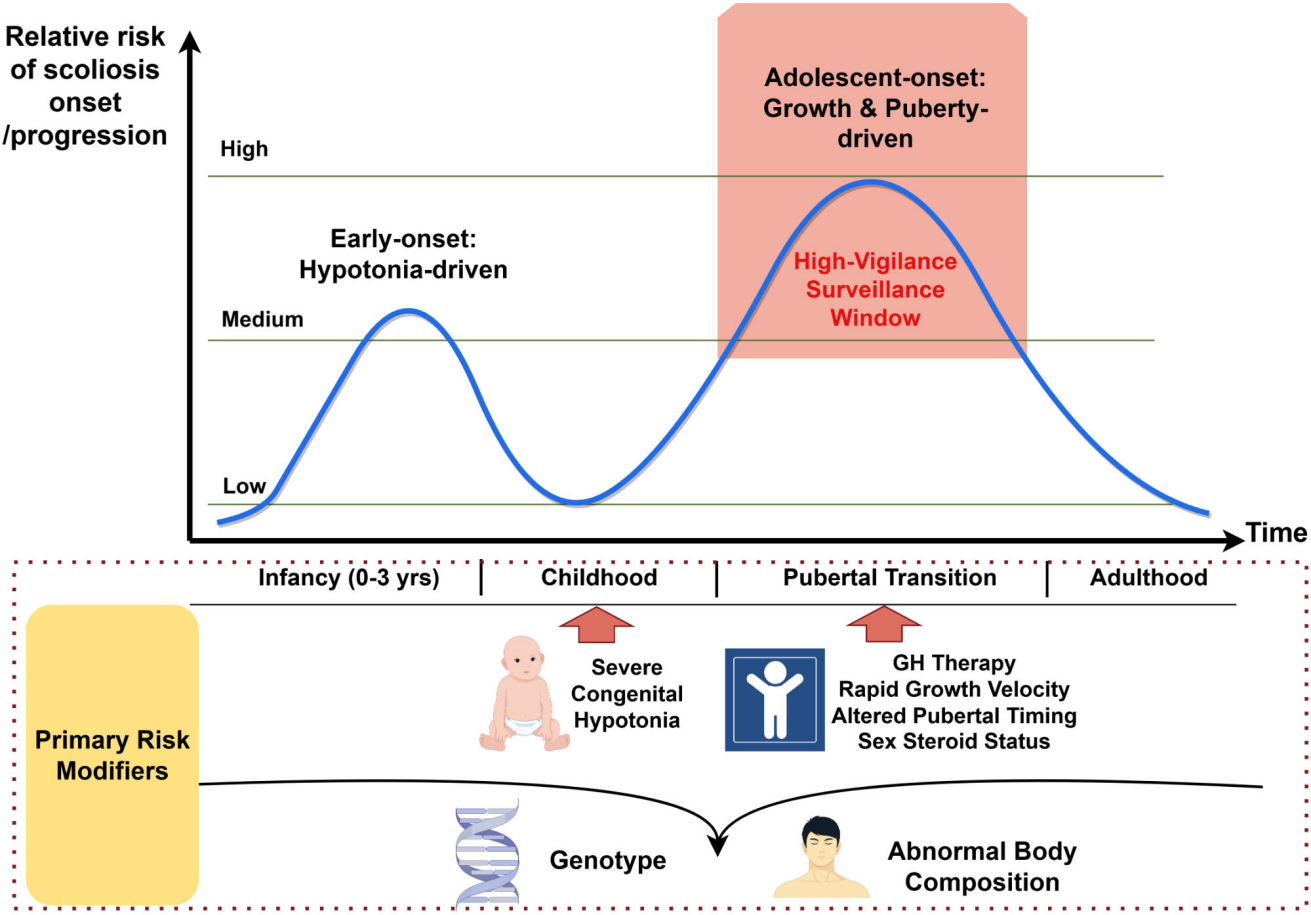
Bimodal risk periods and key modifiers for scoliosis across development in Prader-Willi syndrome. This schematic illustrates the age-dependent, bimodal pattern of scoliosis risk in PWS. The first peak occurs in infancy and is primarily driven by severe congenital hypotonia. The second, more prominent peak coincides with the pubertal transition, driven by the convergence of rapid linear growth (often augmented by GH therapy), altered pubertal timing, and hormonal changes. Key modifiers (e.g., genotype, abnormal body composition) are shown across the developmental timeline, interacting with these critical windows to influence individual risk. The pubertal risk window is highlighted as a period necessitating high-vigilance surveillance. PWS, Prader-Willi syndrome; GH, growth hormone. Image drawn by www.figdraw.com.

The distinct epidemiological, phenotypic, and risk profiles of scoliosis in PWS, which fundamentally differ from those of adolescent idiopathic scoliosis, are systematically compared in **Table 1**. This contrast underscores why surveillance paradigms for idiopathic scoliosis are inadequate for PWS. Furthermore, the cumulative epidemiological evidence delineates two critical, age-dependent high-risk periods—infancy and pubertal transition—as visualized in **Figure 1**. These features provide a compelling rationale for transitioning from uniform, age-based surveillance to the longitudinal, endocrine-informed, risk-stratified monitoring framework proposed in later sections.

**Table 1:** Contrasting features of scoliosis in Prader-Willi syndrome versus adolescent idiopathic scoliosis.

Feature	Scoliosis in Prader-Willi syndrome	Adolescent idiopathic scoliosis	References
Underlying Pathophysiology	Neuroendocrine-driven: Rooted in hypothalamic dysfunction, leading to GH deficiency, hypogonadism, congenital hypotonia, and abnormal body composition. A systemic disorder with spinal manifestations.	Idiopathic/Multifactorial: Presumed genetic predisposition with biomechanical and neuromuscular factors. Primarily a spinal disorder.	(1), (4), (16)
Epidemiology & Natural History	High prevalence (15-80%), cumulative with age. Bimodal onset: peak in infancy (hypotonia-driven) and a second, larger peak during pubertal transition (growth-driven).	Lower population prevalence (~2-3%). Typically presents and progresses during the adolescent growth spurt.	(1), (3), (4), (9), (12)
Key Risk Modifiers	Dynamic endocrine factors: GH therapy status and phase, growth velocity, pubertal stage/timing, sex steroid status. Syndrome-specific factors: Body composition (low lean mass), degree of hypotonia, genotype.	Static/Structural factors: Curve magnitude, skeletal maturity (Risser sign), family history. Growth velocity is a generic risk factor.	(9), (13), (14), (15), (16), (19), (25), (30), (32)
Primary Biomechanical Insult	Combined deficit: Severe muscle weakness (hypotonia) + abnormal spinal loading (altered body composition) → compromised dynamic stability.	Relative imbalance: Asymmetric growth/loading in a neurologically normal spine.	(30), (31), (32)
Implication for Surveillance	Requires endocrine-informed, longitudinal strategy. Intensity must adapt to dynamic risk windows (e.g., GH start, puberty). Chronological age is insufficient. EOS requires distinct orthopedic frameworks.	Largely age- and curve-based strategy (e.g., school screening, follow-up based on Cobb angle and skeletal maturity).	(1), (3), (6), (14), (25)

GH, growth hormone; EOS, early-onset scoliosis. This table synthesizes the fundamental distinctions between scoliosis in PWS and adolescent idiopathic scoliosis, contrasting their underlying pathophysiology, epidemiological patterns, key risk modifiers, and biomechanical origins. These contrasts underscore why surveillance and management paradigms for idiopathic scoliosis are inadequate for PWS and provide the foundational rationale for transitioning to an endocrine-informed monitoring framework.

## 3 Neuroendocrine and biomechanical determinants of scoliosis risk in PWS

### 3.1 Neuroendocrine determinants of scoliosis risk in PWS

#### 3.1.1 Hypothalamic dysfunction and disruption of endocrine axes

PWS is fundamentally characterized by hypothalamic dysfunction, which leads to widespread disruption of multiple endocrine axes, including the GH/insulin-like growth factor-1 (IGF-1) axis, the gonadal axis, and regulators of appetite, metabolism, and energy balance. These endocrine abnormalities play a central role in shaping musculoskeletal development and represent key biological determinants of scoliosis risk in this population. GH deficiency is highly prevalent in PWS and contributes to reduced muscle mass, impaired bone mineralization, and altered vertebral growth patterns, collectively predisposing the spine to mechanical instability. Recombinant human GH therapy partially corrects these deficits by improving lean body mass, muscle strength, and bone density; however, by accelerating longitudinal growth, GH also modifies the temporal dynamics of scoliosis risk, particularly during periods of rapid growth (16–18).

Hypogonadism is another hallmark feature of PWS and results from combined central and peripheral gonadal dysfunction. Sex steroids such as estrogen and testosterone are essential for skeletal maturation, epiphyseal closure, and accrual of muscle mass, all of which contribute to spinal stability. In PWS, delayed or incomplete pubertal development prolongs the period during which the spine remains vulnerable to deforming forces. Inadequate or delayed sex steroid replacement may further compromise bone health and muscular support, thereby extending the window of scoliosis risk into adolescence and early adulthood (19–21). From a monitoring perspective, delayed skeletal maturation implies that chronological age alone is an insufficient guide for surveillance intensity, necessitating integration of pubertal stage and hormonal status into risk assessment.

In addition to GH and gonadal axes, hypothalamic dysfunction in PWS affects other endocrine systems relevant to skeletal health. Central hypothyroidism is reported in up to 30% of patients, and thyroid hormones play a critical role in bone growth and maturation. Adrenal axis abnormalities, although less common, may influence stress responses and perioperative risk. Collectively, these multi-axis endocrine disturbances underscore the need for comprehensive endocrine evaluation when assessing scoliosis risk and progression in PWS (22–24).

#### 3.1.2 Puberty, growth dynamics, and endocrine timing as risk modifiers

In addition to tonic endocrine deficiencies, dynamic changes during puberty represent a critical period for scoliosis progression. Puberty represents a critical inflection point for scoliosis risk in PWS, driven by the convergence of rapid linear growth, hormonal changes, and evolving body composition. Growth velocity during puberty—whether spontaneous or GH-augmented—is a key determinant of curve progression. This phenomenon parallels the “growth acceleration hypothesis” described in adolescent idiopathic scoliosis, yet occurs in a markedly different endocrine milieu in PWS (25). The timing, tempo, and completeness of pubertal development vary substantially among individuals with PWS, ranging from delayed or incomplete puberty to rare cases of central precocious puberty.

Sex steroid exposure during puberty modulates bone mineral density, muscle mass accrual, and epiphyseal closure, all of which influence spinal biomechanics. Studies have demonstrated that lean body mass can increase during spontaneous or induced puberty in GH-treated boys with PWS; however, muscle strength and endurance may remain insufficient to fully stabilize the spine, particularly in the context of persistent hypotonia or inadequate sex steroid replacement (26, 27). Consequently, surveillance intensity must be coupled with pubertal stage and growth velocity, rather than chronological age alone.

#### 3.1.3 Genotype–phenotype modifiers and behavioral factors

Genetic subtype represents an additional modifier of scoliosis risk and monitoring complexity in PWS. Patients with paternal deletions of chromosome 15q11–q13 have been reported to exhibit higher scoliosis prevalence and more pronounced hypotonia compared with those with maternal uniparental disomy, although findings are not entirely consistent across studies (13, 28). Beyond structural risk, genotype is associated with differences in neurobehavioral profiles. Individuals with maternal uniparental disomy more frequently exhibit autism spectrum traits and psychiatric comorbidities, which may adversely affect adherence to physical therapy, brace use, and follow-up schedules (9).

These behavioral and psychosocial factors indirectly influence scoliosis outcomes by modifying the effectiveness of conservative management strategies. As such, genotype should be viewed not as a primary determinant of scoliosis onset, but as a contextual factor that shapes risk stratification, monitoring feasibility, and multidisciplinary care planning.

### 3.2 Biomechanical determinants of scoliosis risk in PWS

#### 3.2.1 Musculoskeletal hypotonia and neuromuscular imbalance

The neuroendocrine abnormalities described above converge on the musculoskeletal system, where hypotonia and neuromuscular imbalance create a biomechanical predisposition to spinal deformity. Congenital hypotonia is a hallmark of PWS and plays a fundamental role in the pathogenesis of scoliosis. Persistent weakness and reduced endurance of trunk and paraspinal muscles compromise dynamic spinal stability, rendering the spine susceptible to deformity under physiological loading conditions. Hypotonia-related scoliosis often manifests early in life as long, flexible curves but may evolve into rigid structural deformities if compensatory mechanisms fail (29).

This neuromuscular imbalance also contributes to the development of hyperkyphosis, as weak paraspinal extensor muscles are unable to maintain an upright posture against gravitational forces and the anteriorly shifted center of gravity, further reinforcing the biomechanical cycle leading to a complex three-dimensional deformity.

The interaction between hypotonia and growth-related factors creates a self-reinforcing biomechanical cycle: initial spinal curvature increases mechanical demand on already weakened musculature, which in turn accelerates curve progression. GH therapy and pubertal growth spurts may intensify this cycle by increasing skeletal growth rate and mechanical stress. From a clinical standpoint, the presence of significant hypotonia or delayed motor milestones should prompt earlier and more frequent scoliosis surveillance, particularly during growth acceleration phases.

#### 3.2.2 Body composition, obesity, and altered spinal biomechanics

Compounding the effects of hypotonia, abnormal body composition further alters spinal loading and postural control. Abnormal body composition is a defining feature of PWS and represents a major biomechanical contributor to scoliosis risk. The combination of reduced lean body mass, increased fat mass, and impaired muscle function alters spinal loading and postural control. Central obesity, which predominates in PWS due to hypothalamic dysregulation of appetite and energy expenditure, increases axial loading on the spine and shifts the center of gravity, thereby exacerbating mechanical stress on vertebral structures (30–32).

**Figure 2:**
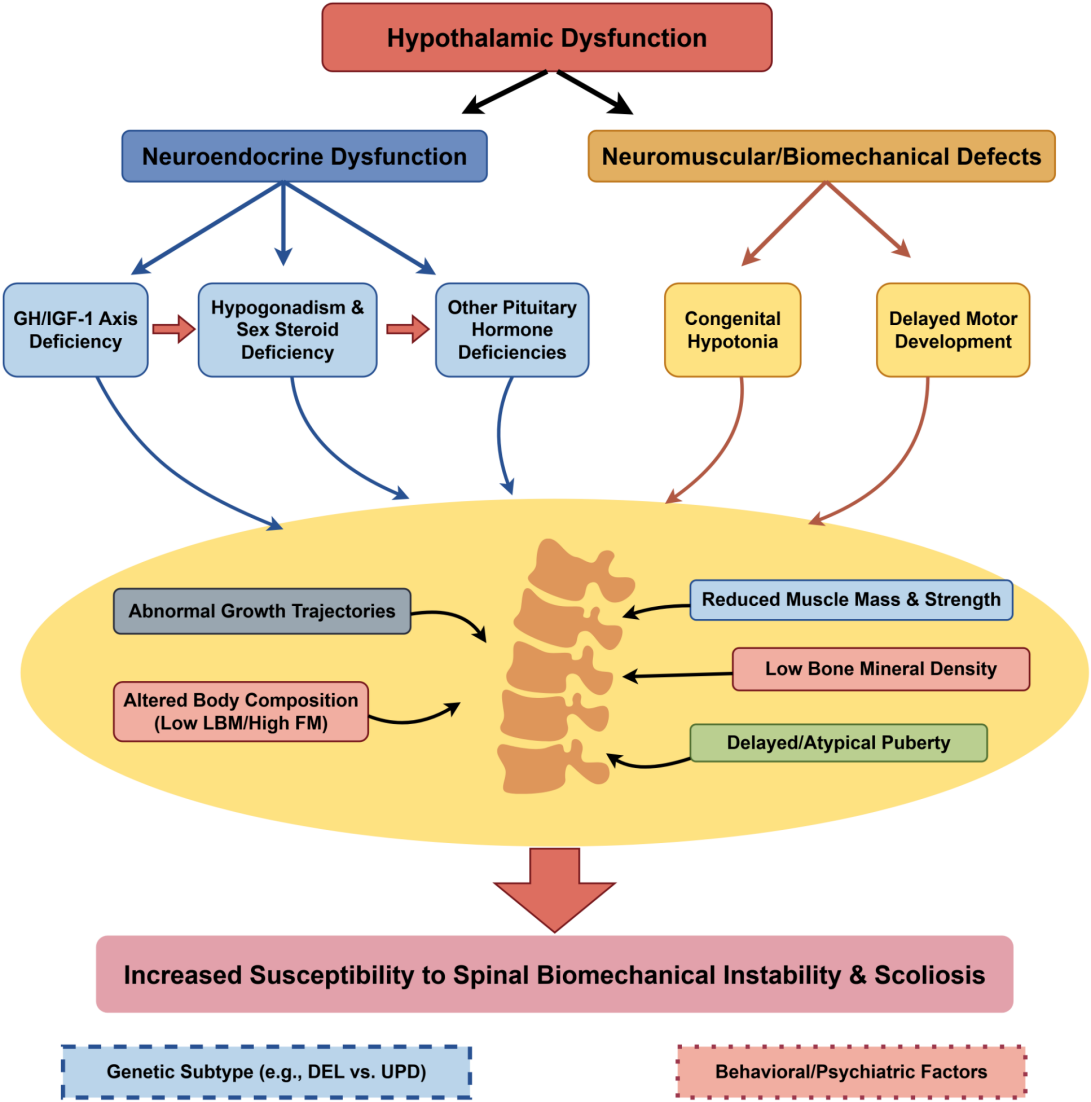
Neuroendocrine and biomechanical determinants of scoliosis risk in Prader-Willi syndrome. This schematic depicts the integrated pathophysiology leading to increased scoliosis susceptibility in PWS. Core hypothalamic dysfunction drives two major pathogenic streams: (1) neuroendocrine disturbances (GH/IGF-1 axis deficiency, hypogonadism) and (2) neuromuscular/biomechanical deficits (congenital hypotonia). These converge to produce intermediate phenotypes, including abnormal growth, altered body composition, reduced muscle strength, and low bone density. Collectively, these factors compromise spinal biomechanical stability. Genetic and behavioral factors act as contextual modifiers across this network. GH, growth hormone; IGF-1, insulin-like growth factor 1; BMI, body mass index; LBM, lean body mass; FM, fat mass; DEL, deletion; UPD, uniparental disomy. Image drawn by www.figdraw.com.

The relationship between obesity and scoliosis in PWS is complex. While some studies suggest that higher body mass index may be associated with a lower prevalence of scoliosis, possibly due to increased passive stability, excess adiposity can impair mobility, balance, and brace tolerance, and may complicate surgical management (33). Importantly, obesity-related biomechanical stress interacts with endocrine-driven growth acceleration, amplifying scoliosis progression risk during puberty. These observations highlight body composition—not BMI alone—as a relevant determinant for risk stratification and monitoring intensity. The complex interplay between neuroendocrine and biomechanical determinants of scoliosis risk in PWS is summarized in **Figure 2**.

## 4 GH therapy as a modifier of scoliosis risk: evidence, controversies, and monitoring implications

### 4.1 Beneficial effects of GH therapy on musculoskeletal development

**Figure 3:**
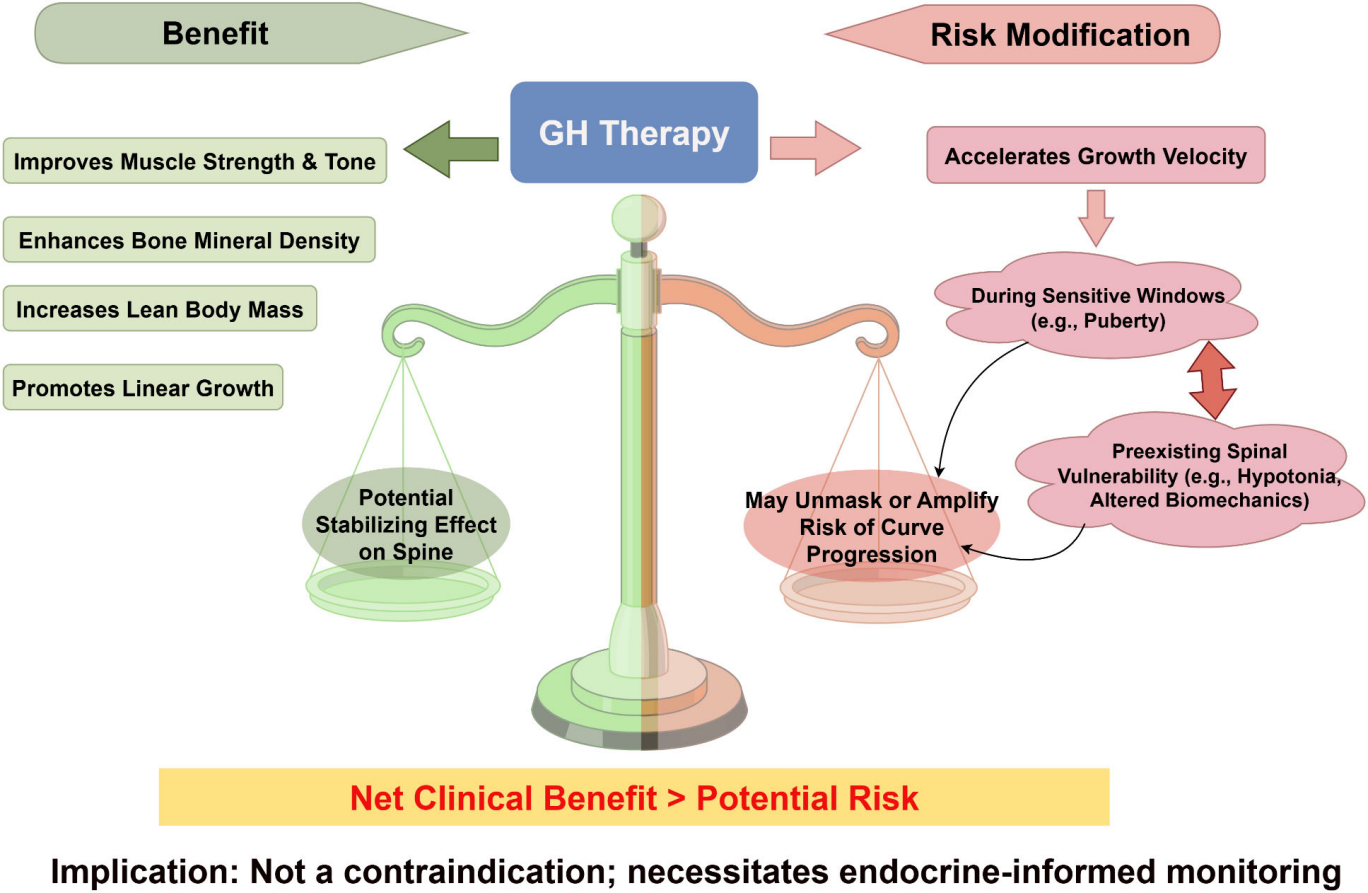
Dual impact of growth hormone therapy on scoliosis risk in Prader-Willi syndrome. This schematic summarizes the balanced effects of GH therapy. Beneficial effects (left) include increased lean mass, improved muscle strength and bone density, and promoted linear growth, which may collectively stabilize the spine. Concurrently, by accelerating growth velocity (right), particularly during sensitive windows like puberty, GH therapy interacts with pre-existing spinal vulnerability (e.g., hypotonia), potentially unmasking or amplifying the risk of curve progression. The net clinical benefit is considered to outweigh the potential risk, supporting GH use with informed monitoring rather than as a contraindication. GH, growth hormone; IGF-1, insulin-like growth factor 1; BMD, bone mineral density. Image drawn by www.figdraw.com.

The complex risk-benefit balance of GH therapy in the context of scoliosis is illustrated in **Figure 3**. GH therapy exerts well-established beneficial effects on growth and body composition in children with PWS and represents a cornerstone of endocrine management. Treatment with recombinant human GH significantly increases linear growth velocity and improves adult height outcomes, addressing the characteristic short stature associated with PWS. Beyond stature, GH therapy induces a favorable shift in body composition by increasing lean body mass and reducing fat mass, as consistently demonstrated by dual-energy X-ray absorptiometry (DXA) and other body composition assessments in pediatric and adult PWS cohorts (34, 35).

Improvements in muscle mass and muscle function are particularly relevant to scoliosis risk. GH therapy has been associated with enhanced muscle tone, increased strength, and improved motor performance, including gains in balance and functional mobility (34). These effects may theoretically contribute to improved spinal stability by augmenting neuromuscular support of the vertebral column. Long-term observational studies further indicate that sustained GH treatment in adults with PWS maintains favorable body composition without increasing cardiovascular or oncological risk, supporting the safety of prolonged therapy (36).

GH therapy also positively influences bone health by increasing bone mineral density (BMD) and modulating bone turnover. Meta-analyses and long-term cohort studies suggest that GH treatment does not adversely affect skeletal integrity and may indirectly protect against scoliosis progression by improving bone quality (35). However, from a monitoring perspective, these musculoskeletal benefits should not be interpreted as eliminating scoliosis risk. Improvements in muscle tone and posture may transiently mask early clinical signs of spinal deformity, underscoring the importance of objective and longitudinal spinal assessment rather than reliance on functional improvement alone during GH therapy.

### 4.2 GH therapy and scoliosis progression: evidence and controversies

Despite its clear systemic benefits, the relationship between GH therapy and scoliosis onset or progression in PWS has been a subject of sustained clinical debate. Case reports and early observational studies noted temporal associations between GH initiation and scoliosis progression, particularly during periods of rapid growth, raising concerns that GH therapy might precipitate or exacerbate spinal deformities. However, larger and more methodologically robust studies have not supported a direct causal relationship.

Longitudinal cohort studies with follow-up durations of up to eight years have demonstrated no significant differences in scoliosis prevalence or severity between GH-treated and untreated individuals with PWS, despite markedly greater height velocity and lean mass accrual in treated patients (14, 15, 37, 38). Similarly, multicenter analyses involving large genetically confirmed PWS cohorts found no significant association between scoliosis and GH treatment status, genotype, pubertal stage, or body mass index (9). These findings suggest that scoliosis progression observed during GH therapy more likely reflects the natural history of PWS intersecting with periods of accelerated growth rather than a direct adverse effect of GH itself. This interaction may be particularly pronounced in individuals with underlying neuromuscular vulnerability, as highlighted in a recent case study of rapidly progressive scoliosis (39).

Evidence from non-PWS populations with short stature has yielded mixed results, with some studies reporting increased scoliosis risk during GH therapy and others finding no significant effect (40–43). While these data highlight the potential role of growth acceleration as a cofactor for curve progression, they also underscore the importance of disease-specific context. In PWS, where hypotonia, altered biomechanics, and endocrine abnormalities are already present, GH therapy should be viewed as a modifier of existing risk rather than an independent etiological factor. Notably, data from large post-marketing surveillance studies, including the PATRO Children study, indicate a low incidence of scoliosis events considered possibly related to GH treatment, further supporting its overall safety profile in PWS (44).

### 4.3 Risk–benefit assessment and implications for scoliosis monitoring

The cumulative evidence strongly supports the conclusion that the benefits of GH therapy in PWS outweigh potential risks related to scoliosis progression. However, given the ongoing controversy and the theoretical risk of growth acceleration on a vulnerable spine, the key clinical implication is not to withhold GH, but to implement enhanced, proactive scoliosis surveillance during GH therapy, particularly at initiation and during puberty. GH treatment confers broad improvements in growth, body composition, metabolic health, physical function, and quality of life, addressing multiple core features of the syndrome (16, 45). Consequently, the presence or risk of scoliosis should not be considered a contraindication to GH therapy in individuals with PWS.

From a clinical management perspective, the key implication is not whether to initiate GH therapy, but how to adapt scoliosis surveillance in response to GH-related growth dynamics. GH initiation, dose escalation, and periods of pubertal transition should be regarded as high-risk windows requiring enhanced spinal monitoring. Baseline assessment of spinal alignment prior to GH initiation is essential, followed by intensified surveillance during phases of rapid height velocity. Monitoring strategies should integrate growth velocity, pubertal stage, and endocrine treatment parameters rather than relying solely on chronological age or curve magnitude.

Importantly, optimization of bone health and timely management of hypogonadism should be considered integral components of GH-related scoliosis risk mitigation. Adequate sex steroid replacement, vitamin D sufficiency, and maintenance of bone mineral density may attenuate the biomechanical vulnerability associated with accelerated growth. Close collaboration between endocrinologists and orthopedic specialists is therefore critical to ensure that GH therapy is delivered safely within a coordinated, multidisciplinary care framework.

Consequently, the presence or risk of scoliosis should not be considered a contraindication to GH therapy in individuals with PWS. Instead, the key clinical implication is the need to adapt surveillance in response to GH-related growth dynamics. In summary, GH therapy in PWS should be conceptualized as a dynamic modifier of scoliosis risk that necessitates tailored, endocrine-informed surveillance rather than treatment avoidance. This perspective provides a critical bridge between mechanistic understanding and clinical practice and directly informs the risk-stratified monitoring framework proposed in the subsequent section.

## 5 Endocrine-informed risk stratification for scoliosis monitoring in PWS

Scoliosis surveillance in PWS requires a paradigm distinct from that applied to idiopathic scoliosis, reflecting the syndrome’s unique neuroendocrine, biomechanical, and developmental context. As outlined in preceding sections, scoliosis risk in PWS is shaped by dynamic interactions between hypothalamic dysfunction, endocrine treatment, growth velocity, pubertal timing, body composition, and baseline spinal status (1, 9, 16–18). These factors fluctuate across development and cannot be adequately captured by chronological age or Cobb angle alone. Consequently, a risk-stratified approach that integrates endocrine and growth-related variables is essential to guide the intensity and timing of scoliosis monitoring in this population.

### 5.1 Rationale for an endocrine-informed, risk-stratified monitoring approach

Traditional scoliosis surveillance strategies primarily rely on static orthopedic parameters, such as curve magnitude and skeletal maturity, to determine follow-up intervals. While these parameters remain important, they fail to account for endocrine-driven growth acceleration and delayed skeletal maturation characteristic of PWS (3, 4). In particular, GH therapy initiation, dose adjustments, and pubertal transition represent periods during which scoliosis risk may increase independently of baseline curve severity (14–16). Similarly, delayed or incomplete puberty may prolong vulnerability to curve progression beyond the age ranges typically considered high risk in idiopathic scoliosis (19–21, 24, 25).

An endocrine-informed framework therefore emphasizes risk stratification based on dynamic modifiers, allowing surveillance intensity to be adjusted proactively in response to changes in growth velocity, hormonal milieu, and body composition. This approach aims to shift scoliosis monitoring in PWS from a reactive, curve-based model to a longitudinal, developmentally informed strategy that anticipates periods of heightened risk (1, 12).

### 5.2 Core variables informing scoliosis risk stratification

Risk stratification in PWS should incorporate a set of core variables that collectively reflect spinal vulnerability at a given developmental stage. These variables can be broadly categorized into endocrine, growth-related, and biomechanical domains.

Endocrine variables include GH treatment status (not treated, initiation phase, stable treatment, or dose escalation), circulating IGF-1 levels within recommended targets, pubertal stage as assessed by Tanner staging, and exposure to sex steroid replacement therapy (16–21). Growth-related variables encompass height velocity, crossing of growth percentiles, and tempo of pubertal progression, and skeletal maturity as assessed by hand X-ray (e.g., Sanders skeletal maturity score) or pelvic radiograph (Risser score, triradiate cartilage closure), which are standard orthopedic tools for timing interventions (46). All of these have been associated with scoliosis onset and progression in PWS and other growth disorders (25, 26). Biomechanical and structural variables include baseline Cobb angle, curve pattern, body composition (lean mass versus fat mass), degree of hypotonia, and functional motor status (3, 30–33).

Genotype and neurobehavioral characteristics further modify risk by influencing hypotonia severity, adherence to conservative treatments, and feasibility of regular follow-up. Patients with paternal deletions and maternal uniparental disomy differ in neuromuscular and behavioral profiles, which may indirectly affect scoliosis outcomes and monitoring feasibility (9, 13, 28).

Importantly, many of these variables are dynamic rather than static. For example, a child with minimal spinal curvature may transiently enter a high-risk state during GH initiation or pubertal growth acceleration, whereas a patient with a stable moderate curve but slow growth velocity may warrant less intensive monitoring. Recognizing this temporal variability is central to effective risk stratification (14, 15). These core variables form the essential inputs for dynamic risk assessment and are detailed in **Table 2**.

**Table 2:** Core variables for endocrine-informed risk stratification of scoliosis in Prader-Willi syndrome.

Domain	Variable	Assessment method	Relevance to scoliosis risk & monitoring	References
Endocrine & Growth	GH Therapy Status & Phase	Clinical history (naïve, initiation, stable, dose escalation)	Dynamic modifier. Initiation/escalation and concurrent puberty create high-risk windows for progression.	(14), (15), (25)
	Growth Velocity	Serial height measurements (cm/year), growth charts; arm span or ulnar length should be used in children with significant scoliosis	Key driver. Rapid velocity, regardless of cause (GH therapy, puberty), is a major risk factor for curve progression.	(1), (25)
	Pubertal Stage	Tanner staging, assessment of secondary sexual characteristics	Critical determinant. Delays/incompleteness prolong spinal vulnerability. Entry into puberty signals need for intensified surveillance.	(4), (19), (25), (26)
	Sex Steroid Status	Hormone levels (LH, FSH, testosterone/estradiol), history of replacement	Influences bone density, muscle mass, and epiphyseal closure. Deficiency may exacerbate risk.	(19), (21), (26)
Spinal & Structural	Baseline Cobb Angle	Standing full-spine radiograph	Cardinal severity measure. ≥20° signifies high risk; ≥10° confirms scoliosis. For early-onset scoliosis, progression thresholds are more aggressive and should be guided by the EOS-C system.	(1), (9)
	Sagittal Profile	Standing full-spine radiograph (measurement of thoracic kyphosis)	Identifies kyphoscoliosis, which adds morbidity from sagittal imbalance. Increased kyphosis alters biomechanical loading and may influence management.	(11)
	Curve Progression Rate	Change in Cobb angle over time (e.g., ≥5°/6-12 months). This definition should be interpreted with caution in EOS, where progression rates can be more aggressive and inform different treatment pathways according to the EOS-C system.	More important than absolute angle in adolescents. Rapid progression mandates escalation of care. In EOS, progression rates are often more aggressive and inform different treatment pathways according to the EOS-C system.	(9)
	Trunk Asymmetry	Clinical exam (Adam‘s forward bend), scoliometer	Screening and monitoring tool. Increasing asymmetry warrants imaging.	(1), (12)
Individual & Syndromic	Body Composition (Lean vs. Fat Mass)	DXA, bioelectrical impedance	Biomechanical modulator. Low lean mass reduces muscular spinal support; high fat mass increases axial load.	(30), (31), (32)
	Degree of Hypotonia	Neuromuscular exam, motor milestone history	Foundational risk factor. Severe hypotonia warrants earlier and more vigilant surveillance, especially in infancy.	(1), (29)
	Genotype	Genetic testing (DEL, UPD, etc.)	Contextual modifier. May influence hypotonia severity, behavioral phenotype (affecting adherence), and possibly prevalence.	(9), (13), (28)

GH, growth hormone; DXA, dual-energy X-ray absorptiometry; DEL, deletion; UPD, uniparental disomy; LH, luteinizing hormone; FSH, follicle-stimulating hormone; EOS, early-onset scoliosis; EOS-C, Early-Onset Scoliosis Classification.

This table provides a systematic compilation of the dynamic and static variables that are essential for endocrine-informed risk stratification of scoliosis in PWS. By categorizing these core determinants—spanning endocrine/growth, spinal/structural, and individual/syndromic domains—it operationalizes the multifactorial risk assessment that is central to the proposed monitoring framework, moving beyond isolated orthopedic metrics.

### 5.3 Proposed risk categories for scoliosis surveillance

**Figure 4:**
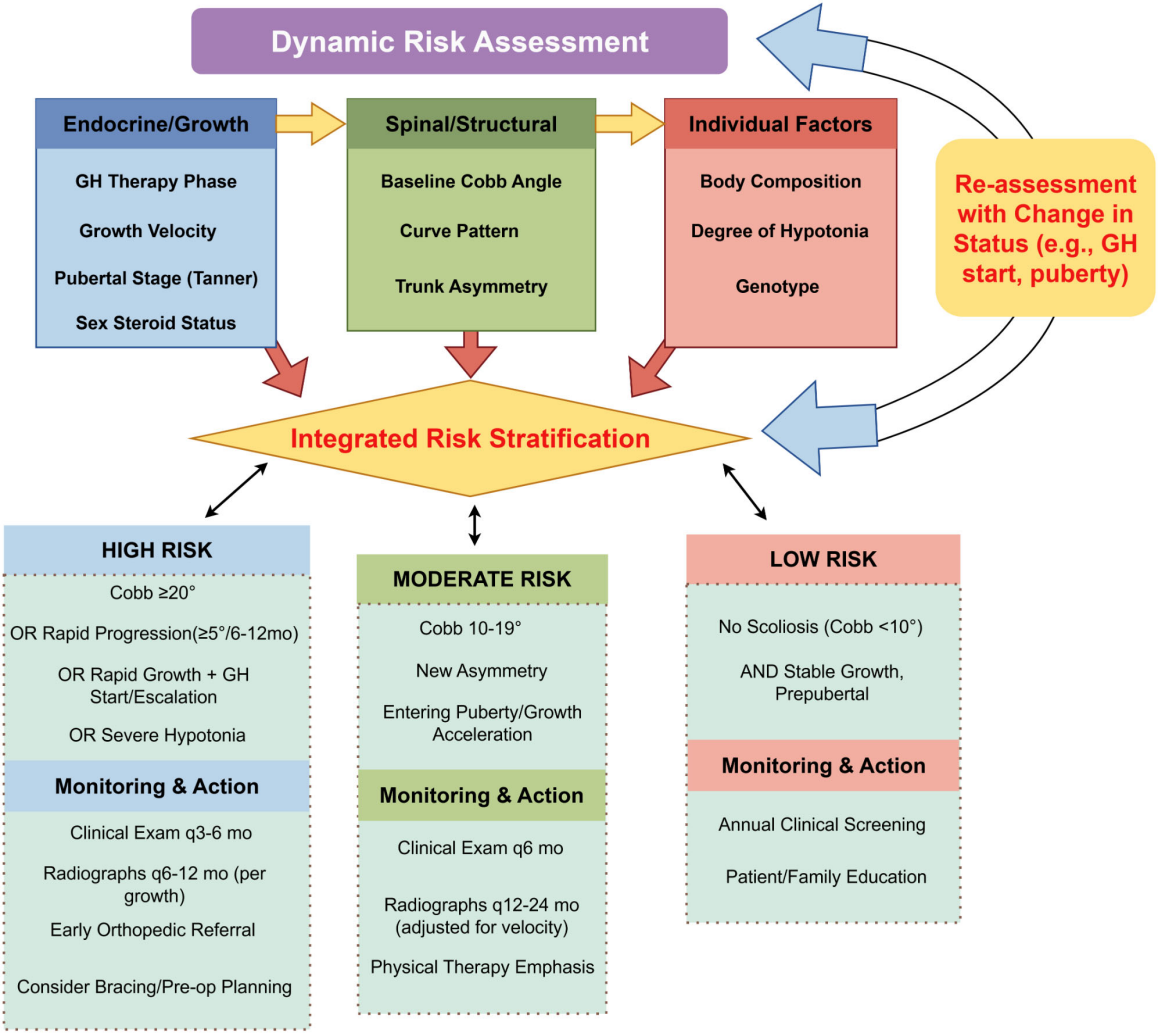
An endocrine-informed, risk-stratified framework for scoliosis monitoring in Prader-Willi syndrome. This clinical decision algorithm proposes a dynamic monitoring framework. It integrates inputs from endocrine/growth status, spinal parameters, and individual factors to perform continuous risk stratification. Patients are categorized into low, moderate, or high-risk states, which dictate specific recommendations for surveillance intensity (clinical and radiographic follow-up frequency) and clinical actions (e.g., physical therapy emphasis, orthopedic referral). The circular arrow emphasizes the need for re-assessment whenever key status changes (e.g., GH initiation, puberty onset), ensuring monitoring aligns with individual developmental trajectories. Proposed framework requiring clinical validation. GH, growth hormone; Cobb, Cobb angle; mo, months. Image drawn by www.figdraw.com. Crucially, the risk thresholds, particularly the Cobb angle criteria, must be interpreted within the context of age of onset. For early-onset scoliosis (onset <10 years), progression thresholds are more aggressive and should be guided by the EOS-C system and evaluated by a specialist, rather than relying solely on the >20° threshold indicated here, which is primarily informative for adolescent-onset curves.

Based on the integration of these variables, individuals with PWS can be broadly classified into low-, moderate-, or high-risk categories for scoliosis progression, with corresponding implications for surveillance intensity (**Figure 4**).

Low-risk patients include those without clinical or radiographic evidence of scoliosis (Cobb angle <10°), stable growth velocity, prepubertal status without imminent growth acceleration, and no recent initiation or escalation of GH therapy (1, 9). In this group, routine clinical examination at standard follow-up visits is generally sufficient, with imaging reserved for emerging clinical asymmetry.

Moderate-risk patients include individuals with mild scoliosis (Cobb angle 10–19°), increasing trunk asymmetry, initiation or early phases of GH therapy, entry into puberty, or rising growth velocity (14, 15, 25). In these patients, structured clinical assessments combined with periodic imaging are warranted, with surveillance intervals adjusted according to growth dynamics rather than age alone.

High-risk patients comprise those with moderate-to-severe scoliosis (Cobb angle ≥20°for adolescent-onset curves; for EOS in infants and young children, any documented progression or a curve exceeding a lower, clinically significant threshold as defined by the EOS-C system should be considered high-risk), documented curve progression, rapid height velocity during puberty, recent GH dose escalation, severe hypotonia, or compromised bone health (9, 14, 29, 47). In this group, intensified monitoring is indicated, including more frequent clinical evaluations, shorter imaging intervals during growth acceleration, and early orthopedic referral when progression thresholds are met (1, 6).

This stratified approach allows patients to move flexibly between risk categories over time, reflecting changes in endocrine treatment, growth patterns, and spinal status rather than fixed classifications.

### 5.4 A practical endocrine-informed monitoring framework

It is important to note that this proposed risk stratification is a conceptual framework based on current evidence. Its clinical utility and validity in predicting progression warrant prospective evaluation in future longitudinal studies to refine thresholds and confirm its value in guiding care. Building on the variables outlined in **Table 2**, we propose an endocrine-informed, risk-stratified scoliosis monitoring framework for children and adolescents with PWS, as depicted in **Figure 4**. This framework integrates the key risk inputs, the process of dynamic stratification, and corresponding surveillance responses. The framework emphasizes two critical high-risk periods—infancy and pubertal transition—and highlights GH therapy initiation and pubertal progression as modifiers that may temporarily escalate monitoring needs (1, 4, 12, 25). By linking risk inputs to actionable monitoring strategies, the framework provides a visual and practical tool to support clinical decision-making and interdisciplinary communication.

### 5.5 Clinical implications for surveillance planning

Implementation of an endocrine-informed risk stratification model has several practical implications. First, baseline spinal assessment should be performed at the time of PWS diagnosis and prior to GH initiation, establishing a reference for future monitoring (1, 9). Second, surveillance intervals should be dynamically adjusted in response to changes in growth velocity, GH treatment phase, and pubertal status, rather than fixed schedules (14, 15, 25). Importantly, when monitoring growth, arm span or ulnar length should be used as a surrogate for height in children with significant scoliosis, as height measurements are confounded by the spinal deformity. Third, endocrine and orthopedic teams should coordinate surveillance planning, ensuring that endocrine treatment decisions are informed by spinal status and vice versa (16, 17).

It is imperative to recognize that the proposed framework, while integrating endocrine factors, must be applied with nuance based on the age of onset. The natural history and management of EOS differ fundamentally from adolescent scoliosis. Therefore, for infants and young children, the threshold for defining ‘high-risk’ is lower and more complex. In this population, any sign of progression, curve rigidity, or a curve magnitude that compromises thoracic development warrants immediate referral to a specialized pediatric orthopedic surgeon with expertise in EOS. The Cobb angle threshold of 20° is retained in the figure as a general benchmark for older children but should not be rigidly applied to infants. Prospective validation of age-specific thresholds within this framework is a critical area for future research.

Importantly, this framework does not advocate for increased imaging indiscriminately but rather supports targeted intensification of monitoring during defined high-risk windows, with consideration of radiation-sparing modalities where appropriate (48, 49). Such an approach balances early detection of progression with minimization of unnecessary imaging exposure.

### 5.6 Summary and transition to monitoring tools

In summary, scoliosis monitoring in PWS is optimally guided by a risk-stratified approach that integrates endocrine, growth-related, and biomechanical determinants of progression (1, 9, 16–18). By recognizing GH therapy and pubertal transition as dynamic modifiers of risk, clinicians can tailor surveillance intensity to individual developmental trajectories. This endocrine-informed stratification model forms the conceptual foundation for evidence-based monitoring strategies and tools, which are discussed in the following section.

## 6 Evidence-based monitoring strategies and tools for scoliosis in PWS

To translate the risk stratification model outlined in **Figure 4** into clinical practice, **Table 3** provides a practical, risk-stratified guide that aligns surveillance intensity, imaging strategy, and multidisciplinary actions with an individual’s risk category. This guide integrates the core variables from **Table 2** and translates them into actionable recommendations for each risk stratum, with specific considerations for the unique challenges of early-onset scoliosis noted in the table footnotes.

**Table 3:** Endocrine-informed monitoring and intervention guide based on risk stratification.

Risk category & criteria	Clinical surveillance	Imaging strategy	Multidisciplinary actions & considerations	References
LOW RISK Cobb angle <10°. No trunk asymmetry. Stable growth velocity. Prepubertal. No imminent GH start.	Annual clinical exam with forward bend test and scoliometer.	Radiographs only if new clinical signs emerge.	Endocrine focus: Routine care. Orthopedic focus: Patient/family education on signs of scoliosis.	(1), (9)
MODERATE RISK Cobb angle 10°–19°. OR New/minimal asymmetry + Dynamic Risk Factor*.	Every 6 months clinical exam. Intensity to every 4 months during active risk phases (e.g., first year of GH, peak puberty).	Baseline radiograph if new diagnosis. Monitoring: Every 1-2 years, shortened to 1 year during rapid growth. Consider radiation-free modalities (ultrasound, topography) for interim checks.	Endocrine focus: Communicate with orthopedics re: GH/puberty timing. Optimize bone health (Vitamin D). Orthopedic/Rehab: Formal physical therapy prescription to strengthen trunk. Consider brace evaluation if approaching 25° in growing child.	(1), (9), (10), (14), (15), (16), (48), (49)
HIGH RISK Cobb angle ≥20°. OR Significant kyphosis / sagittal imbalance. OR Documented rapid progression (≥5°/6-12mo). OR Severe hypotonia + growth spurt. OR Moderate curve + Dynamic Risk Factor*.	Every 3-4 months clinical exam. Very close monitoring during pubertal growth spurt.	Frequent monitoring: Radiographs every 6-12 months based on growth velocity. MRI if atypical features (early onset, neurologic signs).	Endocrine-Orthopedic Coordination: Essential. Joint decision-making on GH dosing/timing vs. surgical planning. Intervention: High likelihood of bracing (Cobb 25°–40°) or surgical evaluation (Cobb >40°–50°, or progressive). Pre-operative endocrine optimization (nutrition, bone health).	(1), (6), (9), (10), (14), (15), (16), (25), (48), (51), (52), (53)

*Dynamic Risk Factors: GH therapy initiation or dose escalation; entry into/active progression through puberty (Tanner stage 2–4); rapid linear growth velocity (14, 15, 25). *Crucially, for infants and young children with early-onset scoliosis (EOS), the "High Risk" threshold is lower and more complex than the Cobb angle criteria indicated here. Any progression, curve rigidity, or thoracic compromise in EOS warrants immediate specialized referral, as guided by the EOS-C system (3, 4). GH, growth hormone; MRI, magnetic resonance imaging.

This table translates the endocrine-informed risk stratification model into a practical, actionable guide for clinical surveillance and intervention in PWS. It defines clear risk categories based on integrated variables, specifies corresponding monitoring intensity and imaging strategies, and outlines stage-appropriate multidisciplinary actions, thereby bridging the proposed framework with direct clinical application.

### 6.1 Clinical monitoring: components and follow-up frequency

Clinical examination represents the foundation of initial scoliosis screening in individuals with PWS and should be performed routinely by the endocrinologist or primary care provider. Any positive findings or high-risk status should prompt timely referral to a spine surgeon for definitive diagnosis and management. Early baseline assessment is particularly important given the high prevalence of early-onset scoliosis and the frequent presence of hypotonia in infancy and early childhood (1, 9). Clinical monitoring should include inspection for shoulder and pelvic asymmetry, evaluation of trunk balance, and the Adam’s forward bend test to detect rotational deformity. Quantitative assessment of trunk rotation using a scoliometer provides an objective measure that facilitates longitudinal comparison over time (1, 12).

Follow-up frequency should be tailored to the patient’s risk category as defined by the endocrine-informed stratification framework. During periods of rapid growth—such as infancy, initiation or escalation of GH therapy, and pubertal transition—clinical examinations are recommended at shorter intervals, typically every 4–6 months (1, 9, 14). In contrast, patients in low-risk states with stable growth velocity and no evidence of scoliosis may be monitored annually. Importantly, neurological examination should be incorporated into routine assessment to exclude underlying neuromuscular or spinal cord pathology that may influence scoliosis progression and management (6).

### 6.2 Imaging considerations

Imaging strategies, including the choice between radiography and emerging non-ionizing modalities, as well as the determination of follow-up intervals, are best determined by the orthopedic surgeon based on individual patient factors, curve characteristics, and skeletal maturity. The role of the endocrinologist is to communicate the patient’s dynamic risk profile (GH status, pubertal stage, growth velocity) to inform the surgeon’s decision-making.

In summary, scoliosis monitoring in PWS should be based on a multimodal strategy that integrates clinical assessment, judicious use of radiographic imaging, and emerging non-ionizing tools within an endocrine-informed, risk-stratified framework. Aligning surveillance intensity with developmental stage, growth dynamics, and endocrine treatment status enhances the clinical utility of monitoring and supports timely intervention. These strategies provide the practical foundation for translating the proposed risk stratification model into routine clinical care and multidisciplinary practice.

## 7 Endocrinology and orthopedics collaboration: seamless transition from monitoring to intervention

### 7.1 Role of conservative management in a collaborative framework

Conservative management remains the first-line approach for scoliosis in individuals with PWS, with physical therapy and bracing serving complementary roles in limiting curve progression. Physical therapy is central to conservative care and should be initiated early, focusing on strengthening trunk musculature, improving postural control, and enhancing functional mobility. Given the pervasive hypotonia and muscle weakness characteristic of PWS, early and individualized physiotherapy is recommended regardless of scoliosis presence, as it may mitigate neuromuscular imbalance and reduce future deformity risk. In patients with mild scoliosis, targeted exercise programs often represent the primary intervention and should emphasize long-term adherence, with strategies adapted to cognitive and behavioral challenges common in PWS (1).

Bracing is typically considered for moderate scoliosis in skeletally immature patients (Risser ≤ 3). However, its application in PWS requires individualized design and close monitoring, with the primary goal of slowing progression rather than achieving full correction (10, 50). Regular follow-up is essential to assess compliance, fit, and curve response. Long-term observational data suggest that carefully monitored bracing, combined with consistent follow-up, can stabilize curves and delay or avoid surgery in selected patients (51).

Despite these strategies, outcomes of conservative treatment in PWS remain heterogeneous. Retrospective studies indicate that a substantial proportion of patients experience progression despite conservative management, highlighting the intrinsic complexity of scoliosis in this population (6). Serial spinal casting may serve as an adjunct or alternative in infants and young children with early-onset scoliosis, as determined by the orthopedic surgeon (4).

### 7.2 Endocrine-orthopedic collaboration: from shared information to coordinated care

Effective management of scoliosis in PWS depends on close integration between endocrine and orthopedic care throughout the disease course. Endocrinologists must remain informed of spinal status when initiating or adjusting GH therapy or managing pubertal induction, as these interventions directly influence growth velocity, bone metabolism, and scoliosis risk. Conversely, orthopedic decision-making should account for GH treatment phase, pubertal stage, and endocrine comorbidities to appropriately time imaging, conservative measures, or surgical intervention.

An integrated, risk-stratified management approach incorporates genetic subtype, GH treatment status, pubertal stage, growth dynamics, baseline Cobb angle, and body composition to guide both monitoring and intervention intensity. Endocrinologists should proactively communicate with the orthopedic team at key decision points, such as: Prior to GH initiation or dose escalation, to ensure baseline spinal assessment has been performed; When planning pubertal induction, to discuss the potential impact on growth velocity and curve progression; If rapid growth or curve progression is suspected during routine endocrine follow-up, to trigger earlier orthopedic referral; To share bone health assessments (e.g., DXA results) that may inform surgical planning or perioperative management.

For example, a patient entering puberty shortly after GH initiation with a mild but evolving curve may warrant closer surveillance and earlier orthopedic input than a prepubertal patient with stable spinal alignment. Coordinated multidisciplinary care also facilitates implementation of supportive measures—including vitamin D supplementation, nutritional optimization, and physiotherapy—that enhance musculoskeletal health and surgical readiness when needed (12, 16, 17, 52, 53).

In summary, transitioning from monitoring to intervention in PWS-associated scoliosis requires a flexible, individualized strategy grounded in endocrine–orthopedic collaboration. Aligning treatment decisions with developmental stage and endocrine context enables timely escalation of care, minimizes avoidable complications, and supports improved long-term musculoskeletal outcomes.

## 8 Conclusion

Scoliosis represents a common and clinically significant complication in individuals with PWS, arising from the complex interplay between hypothalamic dysfunction, endocrine abnormalities, congenital hypotonia, altered body composition, and atypical growth trajectories. Unlike idiopathic scoliosis, spinal deformity in PWS follows a distinct developmental course characterized by two critical high-risk windows—infancy and pubertal transition—during which curve onset and progression are most likely to occur.

This review highlights GH therapy and pubertal development as central modifiers of scoliosis risk rather than independent causative factors. GH therapy provides substantial benefits for growth, body composition, muscle strength, and overall health in PWS and should not be withheld because of scoliosis concerns. Instead, GH initiation, dose escalation, and periods of accelerated growth should be recognized as phases requiring enhanced and proactive spinal monitoring. Similarly, delayed or incomplete pubertal maturation may prolong vulnerability to curve progression, underscoring the importance of integrating pubertal stage and hormonal status into surveillance planning.

By synthesizing epidemiological evidence, mechanistic insights, and clinical data, we propose an endocrine-informed, risk-stratified framework for scoliosis monitoring in children and adolescents with PWS. This framework integrates growth dynamics, endocrine treatment parameters, pubertal status, body composition, genotype-related modifiers, and baseline spinal characteristics to guide surveillance intensity and imaging strategies. Importantly, it allows for dynamic reassignment of risk categories over time, aligning monitoring practices with individual developmental trajectories rather than fixed age- or curve-based thresholds.

The practical implementation of this model emphasizes coordinated, multidisciplinary care between endocrinologists and orthopedic specialists. Several gaps remain that warrant future investigation, including prospective validation of the proposed risk stratification model, standardization of outcome measures, and further validation of non-ionizing imaging tools in PWS populations. Adoption of this framework has the potential to improve early detection and optimize intervention timing for scoliosis, ultimately enhancing long-term health and quality-of-life outcomes for individuals with PWS.
